# Expanded spectrum of exon 33 and 34 mutations in *SRCAP* and follow-up in patients with Floating-Harbor syndrome

**DOI:** 10.1186/s12881-014-0127-0

**Published:** 2014-11-30

**Authors:** Wenke Seifert, Peter Meinecke, Gabriele Krüger, Eva Rossier, Wolfram Heinritz, Achim Wüsthof, Denise Horn

**Affiliations:** Institut für Vegetative Anatomie, Charité - Universitätsmedizin Berlin, Berlin, Germany; Institut für Humangenetik, Universitätsklinikum Hamburg-Eppendorf, Hamburg, Germany; Institut für Medizinische Genetik, Universität Rostock, Rostock, Germany; Genetikum Stuttgart, Stuttgart, Germany; Praxis für Humangenetik Cottbus, Cottbus, Germany; Endokrinologikum Hamburg, Hamburg, Germany; Institut für Medizinische Genetik und Humangenetik, Charité - Universitätsmedizin Berlin, Augustenburger Platz 1, 13353 Berlin, Germany

**Keywords:** SRCAP, Floating-Harbor syndrome, Short stature, Growth hormone therapy

## Abstract

**Background:**

Floating-Harbor syndrome is a rare autosomal dominant short stature syndrome with retarded speech development, intellectual disability and dysmorphic facial features. Recently dominant mutations almost exclusively located in exon 34 of the *Snf2-related CREBBP activator protein* gene were identified to cause FHS.

**Methods:**

Here we report the genetic analysis of 5 patients fulfilling the diagnostic criteria of FHS obtained by Sanger sequencing. All of them presented with short stature, speech delay as well as psychomotor delay and typical facial dysmorphism. Three patients showed a good response to growth hormone treatment.

**Results:**

Two patients demonstrate novel, heterozygous *de novo* frameshift mutations in exon 34 (c.7396delA and c.7218dupT) leading to premature stop mutations in *SRCAP* (p.Val2466Tyrfs*9 and p.Gln2407Serfs*36, respectively). In two further patients we found already known *SRCAP* mutations in exon 34, c.7330C > T and c.7303C > T, respectively, which also lead to premature stop codons: p.Arg2444* and p.Arg2435*. In one patient, we identified a novel *de novo* stop mutation in exon 33 (c.6985C > T, p.Arg2329*) demonstrating that not all FHS cases are caused by mutations in exon 34 of *SRCAP*.

**Conclusions:**

Our data confirm a mutational hot spot in the final exon of *SRCAP* in the majority of FHS patients but also show that exon 33 of this gene can be affected.

## Background

**Figure 1:**
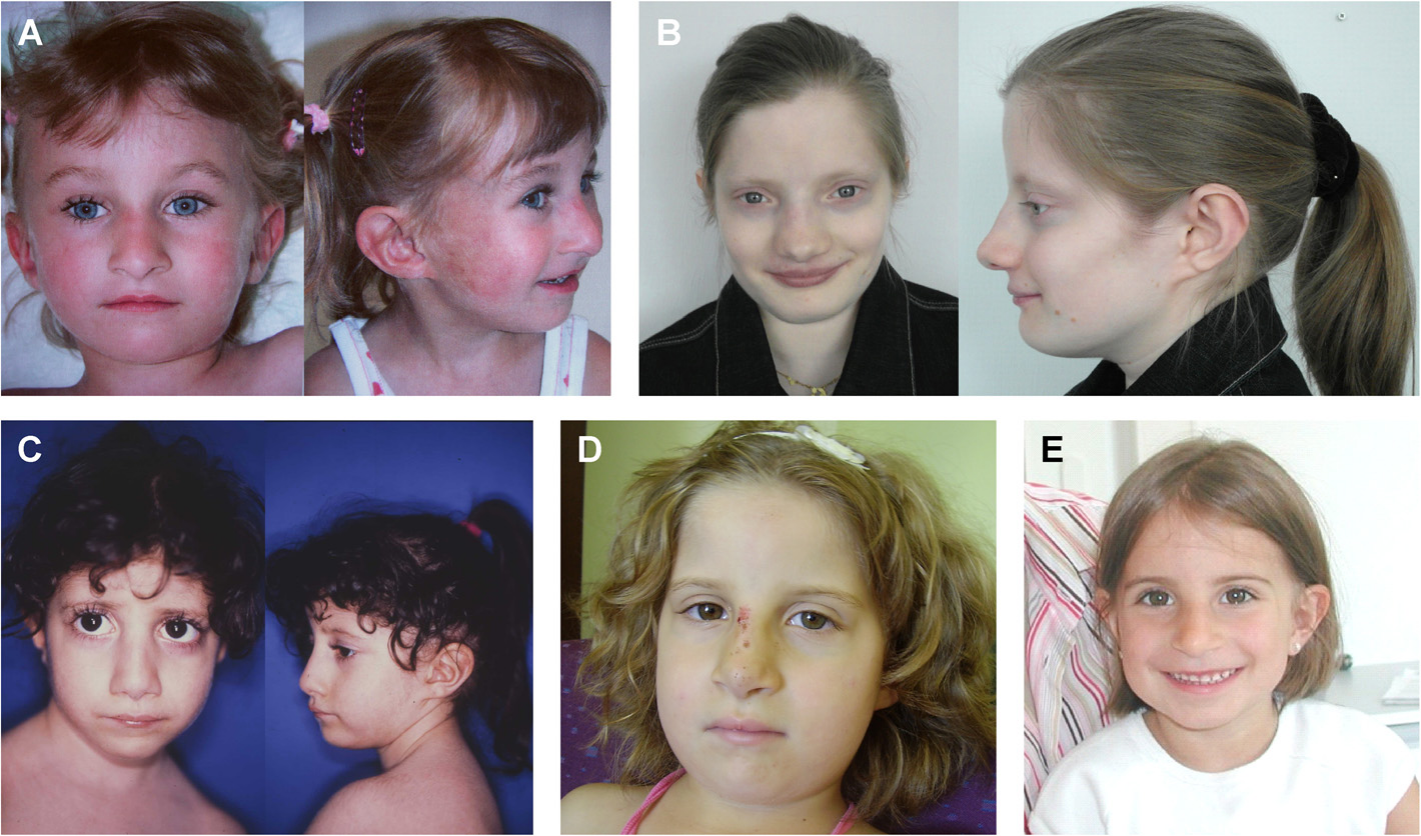
**Photographs of patients, showing facial characteristics of Floating-Harbor syndrome. A** - Patient A at age 5 years. **B** - Patient **B** at 22 years. **C** - Patient C at 5.5 years. **D** - Patient D at 7 years. **E** - Patient E at age of 5.5 years. Note overlapping facial dysmorphisms such as long-hanging columella, short philtrum, and thin lips.

Floating-Harbor syndrome (FHS, OMIM: #136140) is a genetic disorder characterized by short stature, delayed bone age, retarded speech development and intellectual disability (ID) as well as characteristic facial dysmorphisms (Figure 1).

Recently mutations located in exon 34 of the *Snf2-related CREBBP activator protein* (*SRCAP*) gene, encoding the core catalytic component of the multiprotein chromatin-remodeling SRCAP complex, were found to cause FHS in about 50 patients [1–4]. One patient who carried an exon 33 *SRCAP* mutation has been reported [5]. *SRCAP* locates to chromosome 16p11.2, comprises 34 exons and encodes 3230 amino acids.

**Figure 2:**
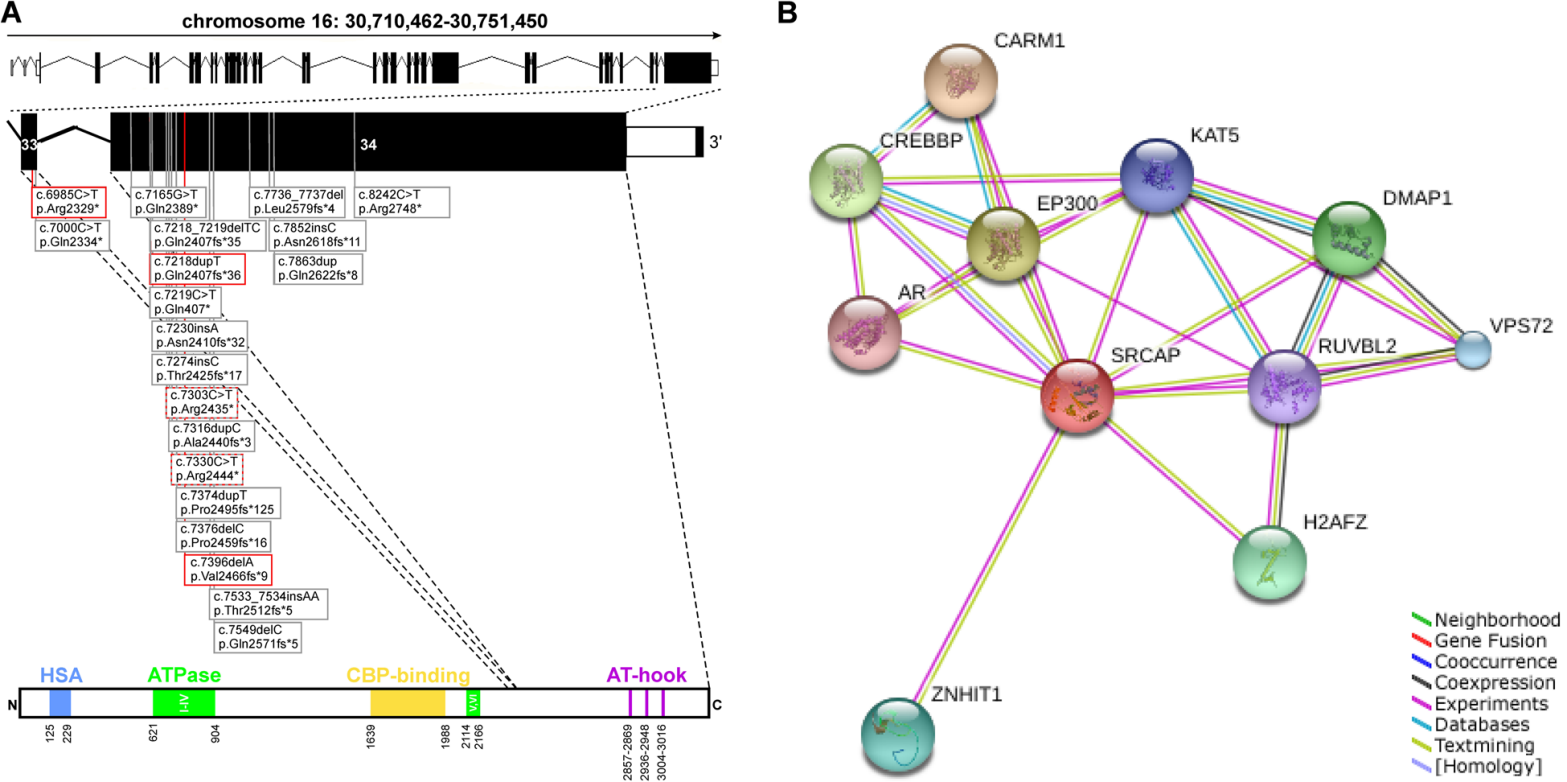
**Schematic representation of the** ***SRCAP*** **gene and positions of known** ***SRCAP*** **mutations. A **- In this study, five *de novo* mutations have been identified to cause FHS (red frame – novel mutations, red-gray dashed frame – recurrent mutations). **B **- Expanded SRCAP protein network predicted functional links to several proteins involved in transcriptional regulation of selected genes by chromatin remodeling including CREBBP.

SRCAP is the catalytic component of the homonymous SRCAP complex which mediates the ATP (adenosine triphosphate)-dependent exchange of a variant histone H2AZ/H2B dimer for a canonical H2A/H2B dimer at nucleosomes, leading to transcriptional regulation of selected genes by chromatin remodeling close to promoter regions. SRCAP is one of several proteins that help to activate a gene called *CREBBP* (Figure 2B). CREBBP plays a key role in regulating cell growth and division and is important for normal development. Mutations in the *SRCAP* gene may result in an altered protein that interferes with normal activation of the *CREBBP* gene, leading to a disturbed development. Rubinstein-Taybi syndrome, an autosomal dominant inherited disorder with some phenotypic overlap, is caused by mutations in the *CREBBP* gene itself [6].

Here we report the clinical and molecular data in 5 patients fulfilling the diagnostic criteria of FHS. All of them presented with short stature, speech as well as psychomotor delay and typical facial dysmorphism including a prominent nose, low-hanging columella and short philtrum.

## Methods

### Patients

Written informed consent forms and permission for publication of this report and accompanying photographs were obtained from all participants or their legal guardians.

The Charité University Medicine ethics board approved this study.

### Mutation analysis

Genomic DNA was isolated from peripheral blood using standard techniques. For mutation screening we amplified the coding region of *SRCAP* [NCBI Reference Sequence: NM_006662.2], including the flanking intronic sequences and the predicted promoter region. Primer sequences and PCR (polymerase chain reaction) conditions are available on request. PCR products were purified using the enzymes exonuclease I and shrimp alkaline phosphatase treatment, and directly sequenced with the BigDye™ Terminator v3.1 Cycle Sequencing Kit (Applied Biosystems) and analyzed on an automated DNA Analyzer (3730 Applied Biosystems).

### Protein network analysis

An expanded protein network of SRCAP was created by the STRING interaction database (string-db.org). As parameters for the network display were used ‘evidence view’, ‘high confidence 0.700’, and ‘no more than 10 interactors’.

## Results

All patients demonstrated heterozygous *de novo* mutations in the gene *SRCAP* (Table 1, Figure 2A). For patients A and B, we identified two novel frameshift mutations in exon 34 (c.7396delA and c.7218dupT, respectively) predicted to introduce premature stop codons in *SRCAP* (p.Val2466Tyrfs*9 and p.Gln2407Serfs*36, respectively). Patients C and D carry previously described point mutations c.7330C > T and c.7303C > T, respectively, which also lead to premature stop codons: p.Arg2444* and p.Arg2435* [1,2]. Interestingly, in patient E, Sanger sequencing identified a novel stop mutation in exon 33 (c.6985C > T, p.Arg2329*) (Figure 2B, red-framed boxes).

**Table 1:** **Summary of clinical and molecular data of patients with Floating-Harbor syndrome**

	**Patient A**	Patient B*	**Patient C**	**Patient D**	**Patient E**
*SRCAP* mutation	Exon 34	Exon34	Exon 34	Exon 34	Exon 33
	c.7395delA (p.Val2466Tyrfs*9)	c.7218dupT (p.Gln2407Serfs*36)	c.7330C > T (p.Arg2444*)	c.7303C > T (p.Arg2435*)	c.6985C > T (p.Arg2329*)
	*de novo*	*de novo*	*de novo*	*de novo*	*de novo*
Sex	Female	Female	Female	Female	Female
Birth weight (SD)	−1	−1	−1.3	−1.3	Mean
Birth length (SD)	−2	−1.8	−1	−1.5	−1
OFC at birth (SD)	−1.5	−1.5	−0.5	n.d.	Mean
Age at first assessment	2 years	2 years	4 years	5 years	5 years 4 months
Short stature (SD)	−2.5	−3.2	−3.6	−2	−3.4
Age at last assessment	10.5 years	22 years	21 years	7 years 3 months	10 years
Height (SD)	−2	154 cm; -1.8	140 cm; -3.7	−2	−1.7
OFC (SD)	−1	−0.7	−1.6	−0.4	−1.7
ID	+	+	+	+	-
Language impairment	+	+	+	+	+
Craniofacial features	Low-hanging columella, short philtrum, thin lips	Broad nasal tip, long columella, short philtrum, thin lips, posteriorly rotated ears	Low-hanging columella, short philtrum, thin lips	Low-hanging columella, short philtrum, thin lips	Prominent nose, low-hanging columella, short philtrum, thin lips
Skeletal anomalies	Delayed bone age, brachydactyly and clinodactyly V	Delayed bone age, clinodactyly V, brachydactyly, broad thumbs	Delayed bone age, broad fingertips, pseudoarthrosis of clavicles	Delayed bone age, brachydactyly V	Broad fingertips, clinodactyly V
Treatment	GH treatment (between the 3^rd^ and the 6^th^ year of life)	GH treatment (between the 5^th^ and the 14^th^ year of life)	-	-	GH treatment
Other findings	Behavioural difficulties	Delayed puberty, primary ovarian insufficiency	Hearing problems, behavioural difficulties, hypermenorrhoea	Hearing loss, microdontia	Behavioural problems

*The clinical manifestations of patient B at age of 5 years were published in the AJMG 10:47-52, 2001.

Table 1 and Figure 1 summarize the clinical data. In the individuals analyzed here birth weights ranged between mean and -1.3 SD (standard deviation) as well as birth lengths between -1 to -2 SD (Table 1). The occipitofrontal head circumference (OFC) at birth was normal in all. Prior puberty bone ages were significantly delayed when X-rays were available (in patients A, B, C, D). Postnatal short stature varied from -1.7 to -3.7 SD, however three patients (patients A, B, E) received growth hormone (GH) therapy during childhood and their heights were between -1.7 to -2 SD at time of last assessment. In all individuals, postnatal OFC was lower than the mean but still in the normal range (-0.4 to -1.7 SD). Delayed pubertal development and primary ovarian insufficiency were observed in patient B. Patient C showed normal pubertal development and hypermenorrhoea in adult age.

Behavioral difficulties such us aggressive behavior, anxiety, sleep disturbances, and rigid mannerisms were observed in two patients starting before and at puberty (patients A and E) and in adulthood (patient C), respectively. Except one, all patients showed delayed speech development as well as reduced cognitive abilities with schooling at schools for mentally handicapped children. As adults two patients were able to speak in short sentences and to read and write with simple skills. Patient E carrying the exon 33 mutation showed only mild speech delay but has normal cognitive skills and attended a normal school.

The characteristic facial aspect with a prominent nose with a broad nasal tip, a low hanging columella, a short philtrum, and a thin upper lip was present in all and remains constant also in adult patients (Figure 1). Minor skeletal abnormalities such us brachydactyly (patients A, B, D), broad fingertips (patients C and E) or pseudoarthrosis of the clavicles (patient C) were observed in all patients studied here.

## Discussion

Our molecular data confirm a mutational hot spot in the final exons of *SRCAP* in all patients tested here. Postnatal short stature with in relation larger OFC together with a distinct facial aspect and delayed speech development as important diagnostic criteria of FHS were fulfilled in all patients reported here. Markedly delayed bone age was disclosed in all patients (A, B, C, D) where hand radiograms were available. Behavioral difficulties were observed in three patients of our study group and occur in about one third in a larger study cohort, therefore behavioral problems should be monitored [4]. Interestingly, growth hormone treatment led to significant growth improvement toward the low normal range in three patients indicating effectiveness of this therapy in patients with FHS. One female patient with the clinical diagnosis of FHS has been reported with precocious puberty following treatment with gonadotrophin-releasing hormone analogue and later growth hormone treatment because of growth hormone deficiency [7]. This patient reached average adult height.

So far unreported endocrinological abnormalities (delayed puberty and primary ovarian insufficiency in patient B and hypermenorrhoea in patient C) were documented in the adult females reported here.

The vast majority of affected individuals carries truncating mutations of exon 34 of *SRCAP* with two mutations (p.Arg2444*, p.Arg2435*) which are recurrently identified (Figure 2A) [1,3,4]. In our cohort we identified these two recurrent mutations and two novel frameshift mutations in exon 34. Moreover, we found one novel mutation in the penultimate exon 33. The phenotype of this patient is in accordance with the manifestations of FHS, except for ID. Interestingly, she is the only one in our cohort with only mild speech delay and normal schooling. One could speculate that this milder phenotype may be due to partial nonsense-mediated decay or faster degradation of the produced altered SRCAP protein. Until now, only one other mutation in exon 33 of *SRCAP* (p.Gln2334*) has been documented in an affected individual with typical features of FHS [5]. Apart from typical manifestations of FHS this 8 year old patient has not only speech delay but also significant intellectual disability in contrast to our patient carrying the exon 33 mutation. However, average intelligence and regular schooling have been already reported in a few affected individuals with exon 34 mutations and therefore the broad range of cognitive skills in patients with FHS seems to include normal psychomotor development [4].

All hitherto FHS causing mutations are predicted to cause a truncated SRCAP protein lacking the putative C-terminal AT-hook DNA binding motif. Due to the observed heredity transmission by heterozygous *de novo* mutation a dominant negative disease mechanism has been postulated [1]. Therefore, truncating mutations outside exons 34 and 33 may result in nonsense mediated decay leading to different phenotypic effects [8]. One example of different phenotypes associated with mutations located in the last exons of a gene versus mutations in other exons is a lipodystrophy-progeroid phenotype in individuals carrying mutations in the last exon of the *FBN1* gene while mutations of other exons of this gene lead to Marfan syndrome [9]. However, absence of *SRCAP* mutations were reported in 3/9 patients investigated by direct sequencing and may have different explanations, e.g. an overlapping phenotypic spectrum with Rubinstein-Taybi syndrome or other syndromes as well as possible genetic heterogeneity in FHS [3]. Potential candidate genes may include proteins in distinct vicinity to SRCAP and CREBBP with functional links to chromatin remodeling mechanisms (Figure 2B). Thus, further work is required to fully elucidate the pathomechanism of FHS.

## Conclusions

In patients with suspected FHS, we strongly recommend that mutational analysis should include not only sequence analysis of exon 34 but should extend to at least exon 33 of this gene. Growth hormone treatment shows effectiveness in patients with FHS.

Endocrinological and gynaecological follow-up is needed for adult patients with FHS to evaluate for further complications.

### Consent

Written informed consent was obtained from the patients/parents for publication of this research and any accompanying images. A copy of the written consent is available for review by the Editor of this journal.
